# The Role of Positron Emission Tomography/Computed Tomography (PET/CT) for Staging and Disease Response Assessment in Localized and Locally Advanced Pancreatic Cancer

**DOI:** 10.3390/cancers13164155

**Published:** 2021-08-18

**Authors:** Michele Ghidini, Marta Vuozzo, Barbara Galassi, Paola Mapelli, Virginia Ceccarossi, Lucio Caccamo, Maria Picchio, Daniele Dondossola

**Affiliations:** 1 Operative Unit of Oncology, Internal Medicine, Fondazione IRCCS Ca’ Granda Ospedale Maggiore Policlinico, 20122 Milan, Italy; barbara.galassi@policlinico.mi.it; 2 Werner Siemens Imaging Center, Department of Preclinical Imaging and Radiopharmacy, Eberhard Karls Universität Tübingen, 72076 Tübingen, Germany; marta.vuozzo@med.uni-tuebingen.de; 3 University Medical Center, Eberhard Karls University Tübingen, 72074 Tübingen, Germany; 4 Vita-Salute San Raffaele University, 20132 Milan, Italy; mapelli.paola@hsr.it (P.M.); picchio.maria@hsr.it (M.P.); 5 Nuclear Medicine Department, IRCCS San Raffaele Scientific Institute, 20132 Milan, Italy; 6 Dipartimento di Chirurgia Generale e dei Trapianti di Fegato, Fondazione IRCCS Ca’ Granda Ospedale Maggiore Policlinico, 20122 Milan, Italy; virginia.ceccarossi@unimi.it (V.C.); lucio.caccamo@policlinico.mi.it (L.C.); daniele.dondossola@policlinico.mi.it (D.D.); 7 Dipartimento di Fisiopatologia Medico-Chirurgica e dei Trapianti, Università degli Studi di Milano, 20122 Milan, Italy

**Keywords:** pancreatic cancer, positron emission tomography, resectable disease, radiomics, ^18^F-FDG, PET tracers

## Abstract

**Simple Summary:**

Pancreatic cancer (PC) has a severe prognosis and even after radical surgery, relapse rate is very high (70–80%). The impact of PET/CT in PC clinical management has been increasingly investigated in the last decades. As regards localized and potentially resectable disease, the role of PET/CT is still controversial and international guidelines do not recommend its routine use. Despite this, PET may play a role in assessing PC stage and grade and potential resectability after neoadjuvant treatment. Aim of this review is to discuss the current use for staging and disease response assessment and future developments of PET/CT in resectable PC.

**Abstract:**

Pancreatic Cancer (PC) has a poor prognosis, with a 5-year survival rate of only 9%. Even after radical surgical procedures, PC patients have poor survival rates, with a high chance of relapse (70–80%). Imaging is involved in all aspects of the clinical management of PC, including detection and characterization of primary tumors and their resectability, assessment of vascular, perineural and lymphatic invasion and detection of distant metastases. The role of Positron Emission Tomography/Computed Tomography (PET/CT) in detecting PC is still controversial, with the international guidelines not recommending its routine use. However, in resectable PC, PET/CT may play a role in assessing PC stage and grade and potential resectability after neoadjuvant treatment. Quantitative image analysis (radiomics) and new PET/CT radiotracers account for future developments in metabolic imaging and may further improve the relevance of this technique in several aspects of PC. In the present review, the current state of the art and future directions of PET/CT in resectable PC are presented.

## 1. Introduction

The correct and timely identification of initial stage of malignancies is of paramount importance to plan therapeutic strategies which might cure the disease, improve disease-free survival and patients’ quality of life. Early diagnosis will drive the choice of definitive, often curative therapy, such as surgery, ablation procedures or 3-dimensional intensity-modulated radiotherapy, preceded by systemic chemotherapy when indicated [1].

A relevant example is the case of pancreatic carcinoma (PC) in which early detection of stage, nodal metastases and occult liver metastases is mandatory to choose the more suitable treatment option. The prognosis of PC is poor, with a 5-year survival rate of only 9% [2]. More than 80% of PC patients suffer from unresectable disease together with distant metastases at diagnosis. A percentage of 74% of patients die within the first year from diagnosis and 94% of deaths occur within 5 years of diagnosis. A median survival of approximately 20 months has been observed for the 10–15% of patients with localized tumors undergoing resection and adjuvant chemotherapy [2]. Significantly better outcomes have been reported for smaller tumors at earlier stages. In patients with localized disease, radical resection determines a 5-year survival rate of 30–60% increasing to 75% for lesions that are less than 10 mm [3]. The main reason of this poor prognosis is that patients are asymptomatic and therefore often diagnosed at advanced stages (80–85%); however, even at early stages, the chance of relapse is high (70–80%) [4]. Surgery is the only curative treatment approach, and an accurate evaluation of resectability is important in order to avoid futile procedures. Therefore, early and accurate diagnosis is essential to determine the most effective therapy [4].

Great improvements have been observed in imaging over the last decade, including Endoscopic Ultrasounds (EUS), Computed Tomography (CT), Magnetic Resonance Imaging (MRI) and Positron Emission Tomography/Computed Tomography (PET/CT). Imaging is crucial in all aspects of the clinical management of PC, from diagnosis and characterization of the pancreatic mass and anatomical variants, description of vascular involvement, perineural and lymphatic invasion to detection of metastases and assessment of resectability [2,5]. In this spectrum, however, the role of imaging with PET/CT in PC is controversial.

This review article will evaluate the current role of PET/CT imaging in PC management.

## 2. ^18^F-Fluorodeoxiglucose (^18^F-FDG) PET/CT for PC Diagnosis

^18^F-flurodeoxyglucose (FDG) PET/CT is used for diagnosis and staging of many malignant diseases. The overall sensitivity of ^18^F-FDG PET/CT ranges between 85% and 97% [6,7,8,9]. However, there is no firm consensus about the role of ^18^F-FDG PET/CT in PC. Peripancreatic nodes may be hidden by ^18^F-FDG uptake in the primary tumors with subsequent low sensitivity of the imaging technique [10,11,12]. Moreover, ^18^F-FDG PET/CT specificity is sometimes reduced because immune cells in tumors and inflammatory lesions uptake the radiotracer as well. Differently, fibroblast-activated protein is rarely expressed in basal conditions and highly expressed on cancer-associated fibroblasts. Therefore, the use of radioactive gallium 68 (^68^Ga) coupled with a fibroblast-activated protein inhibitor (FAPI) is increasingly being evaluated also in PC for higher specificity with respect to traditional ^18^F-FDG PET/CT [13,14]. Some authors reported a better sensitivity of ^18^F-FDG PET/CT compared to CT (42 vs. 35%) [15]. For liver metastases, PET/CT has shown high sensitivity, allowing distinction between malignant and benign lesions [7]. Several studies showed the superiority of ^18^F-FDG PET/CT for detection of distant metastases [15,16,17], while Heinrich et al. reported a change in the disease management driven by PET/CT in 16% of the cases [18]. Despite this, international guidelines do not recommend the routine use of PET/CT. Table 1 reports the latest National Comprehensive Cancer Network (NCCN) [19], European Society of Medical Oncology (ESMO) [20] and Associazione Italiana di Oncologia Medica (AIOM) [21] guidelines for the use of CT, MRI and PET/CT scan in PC.

## 3. The Role of ^18^F-FDG PET/CT in Surgical Management of PC

Even after radical surgical procedures, PC leads to poor results in terms of patient survival. In addition, the low resectability rate (20–30%) at presentation implies the need of an accurate pre-surgical staging. Neoadjuvant Chemotherapy (NAT) is increasingly used with the aim of increasing the rate of curative resections. PET/CT is widely used to define pancreatic local involvement with a sensitivity and specificity of 81–97% and 72–76%, respectively [22,23]. The role of PET/CT is widely investigated owing to its ability to depict the metabolic activity of a lesion and to improve the definition of PC stage during diagnostic phase. PET/CT may have an impact especially on patient’s management prior surgery and/or after NAT, providing a more accurate preoperative staging and predicting the likelihood of pathological treatment response to NAT [24].

In this section we will examine the usefulness of PET in refining diagnostic and therapeutic iter of PC.

### 3.1. The Role of ^18^F-FDG PET/CT in PC Differential Diagnosis

When planning a surgical or medical treatment, an appropriate differential diagnosis between benign and malignant lesions must be achieved. This step could be particularly difficult in PC because different pancreatic lesions can mimic the characteristics of PC. In addition, due to the heterogeneity of the published studies and the type of pancreatic lesions, it is not possible to identify a gold standard diagnostic technique used to differentiate benign and malignant pancreatic lesions. In this context, PET/CT could provide useful information aimed at improving diagnosis considering its high sensitivity but not replacing other imaging techniques due to low specificity [25]. In a retrospective study by Ergul et al., PET/CT showed a sensitivity and specificity of 100% and 89.5% in the differential diagnosis between malignant and benign lesions [26], while in a recent prospective study by Krishnaraju et al., sensitivity and specificity decreased to 89% and 57%, respectively [27]. It has been reported a possible increase of the diagnostic performance of PET/CT by using SUV cut-off and FDG uptake pattern to discriminate benign vs. malignant lesions [28,29]. Indeed, differential diagnosis between PC, pancreatitis-associated inflammation and cystic lesions is often difficult with the use of CT scan. In patients with chronic pancreatitis, risk of PC development is high; therefore, a negative PET/CT examination has a negative predictive value (NPV) of 100% in these cases, while a positive FDG uptake is highly suspicious for neoplastic degeneration [29].

The inclusion of PET/CT into the standard diagnostic iter was able to correctly change the diagnosis for both malignant and non-malignant lesion in 8.3% of patients (diagnosis confirmed at pathological analyses), while in 3.6% of them the diagnosis was incorrectly changed.

While the role of PET/CT is debated, ultrasound guided fine needle aspiration (FNA-EUS) is increasingly used to evaluate the nature of a pancreatic lesion [30]. A retrospective study was carried on by Sant Louis’s group in order to compare the performance of these two techniques. In a group of 25 patients, sensitivity, specificity, positive predictive value (PPV), NPV and accuracy of FNA-EUS in differential diagnosis were 91%, 100%, 100%, 50% and 92%, respectively, while they were 65%, 100%, 100%, 20% and 68% for PET/CT, respectively. The conclusion of the authors was that EUS-FNA should have been preferred in the workup of pancreatic lesions due to its higher sensitivity and accuracy [31]. However, Ergul and colleagues showed very similar diagnostic accuracy in discriminating benign to malignant diseases of PET/CT and EUS/FNA [26]. In a scenario where PET/CT and EUS/FNA have equal accuracy, the improved diagnostic potential of FNA-EUS could be counterbalanced by the possible complications of an invasive technique due to histological sampling [30]. As a result, no definitive conclusion could be drawn and PET/CT or EUS/FNA could be adopted in diagnostic pathway to discriminate benign from malignant lesions according to center expertise and preferences.

### 3.2. Pre-Surgical Tumors Staging and Grading

Preoperative diagnostic iter in case of PC is intended to evaluate the resectability of the primary tumor and obtain a proper tumor staging. These issues are of primary importance because incorrect presurgical staging could result in microscopic positive margins (R1) and macroscopic positive margins (R2) resections or futile explorative laparotomy with a reduction in patients’ quality of life. For these reasons, both local and distant neoplastic involvement must be properly evaluated. Indeed, while patients with distant metastases were generally excluded by a surgical iter, borderline resectable and locally advanced tumors should undergo NAT prior to surgery. The local extension of PC could be evaluated through CT and MRI due to their improved anatomical visualization. EUS could add further information when local and nodal involvement (especially of mesenteric artery and vein) are equivocal due to a direct visualization of pancreatic head and peripancreatic region [25]. Compared to PET/CT, the ability of CT, MRI and EUS to properly define tumors border and local spread was higher [31]. Nevertheless, some interesting perspectives on the role of ^18^F-FDG PET/CT were pointed out by Lai and colleagues [32]. Indeed, they found no significant difference between tumors size at histopathological analyses and tumors size measured at PET/CT, suggesting that PET/CT could provide adequate information on tumors size and volume prior to surgery.

In addition to local involvement, increasing evidence showed that a subset of patients had occult metastatic diseases [33]. The role of PET/CT in improving the staging ability of CT and MRI was evaluated. The high glucose uptake of PC cells enabled the localization of distant metastasis with a sensibility of approximately 90%, higher than CT and MRI [11,18,34]. In a cohort of 50 patients, PET/CT in combination with the other diagnostic techniques showed a sensitivity of 82% in detecting liver metastasis and 80% in detecting peritoneal carcinomatosis. However, in 5 patients liver metastasis were not recognized before surgery [17]. In another study, PET/CT fusion images allowed the identification of 3 unknown lesions and 2 additional pancreatic nodules (all confirmed with histopathology) [35]. A recent meta-analysis confirmed these data with a pooled sensitivity and specificity of 80% (95% CI: 67–89%) and 100% (95% CI: 89–100%), respectively [36]. Interestingly, a reduction in sensitivity was shown together with lesion size (97% in lesions > 1 cm and 43% in lesions < 1 cm) [37].

While PET/CT showed high sensitivity and specificity in detecting distant metastasis, its role in properly evaluating nodal (N) stage is debated. Even though locoregional N+ is not a non-resectability criteria, it could be identified as a main negative prognostic factor and N+ patients should receive NAT. Lemke and colleagues were able to detect 10 N+ patients before surgery among the 31 N+ patients at pathological analysis through the use of contrast enhanced PET/CT [35]. Carbohydrate antigen 19.9 (CA19.9) > 240.55 U/mL and standardized uptake value (SUV) max > 7.05 were used to predict lymph node micro-metastasis and improve the limited sensitivity (about 40%) of imaging per se [38,39]. Indeed, the identification of local lymph node can be difficult due to the high ^18^F-FDG uptake of the primary tumors with a reduction in sensitivity [sensitivity 55% (95% CI: 38–72%); specificity 94% (95% CI: 81–98%)] [36]. In a study carried out by Santhosh and collaborators [38], 37 out of the 54 patients included were considered resectable and underwent surgery. Among them, nodal involvement was detected in 18 cases. PET/CT missed two cases with no false positives, while CT detected only 6 positive cases. The sensitivity, specificity, PPV, NPV and accuracy for nodal staging were 33%, 84%, 67%, 60% and 59% for CT and 89%, 100%, 100%, 90% and 95% for PET/CT, respectively [40].

The performance of PET/CT in modifying cancer staging was assessed in a recent multicenter study. Interestingly, once PC was confined to the peripancreatic tissue (stage < IIB), CT showed an improved accuracy. Conversely, in pathological stage IIB and especially in stage IV, PET/CT was shown to be able to properly modify staging in 21% and 41%, respectively [33].

Together with cancer stage, tumor grade could deeply impact and modify post-surgical results. Hence, quantitative imaging metrics at PET/CT can help in fulfilling this issue. In a recent study, different quantitative parameters were related with PC grade and were able to predict patient survival and progression free survival in operated patients26. Consistently, SUV value max > 3.6 was recently associated to higher tumors grade (*p* = 0.023), worse disease-free (*p* = 0.001) and overall survival (*p* = 0.002). It can be speculated that the identification of patients with high-risk recurrence using PET/CT could modify clinical management-delaying surgical treatment and applying neoadjuvant chemotherapeutic protocols. This conclusion was suggested also by Pergolini and colleagues who identified a subgroup of patients (SUV max ≥ 6.0 with CA 19.9 ≥ 200 U/mL) with lower disease-free survival after surgery and that could benefit of a systemic treatment before surgery [41]. These data were recently supported by Moon and colleagues who identify a CA19.9 > 150 U/mL and SUV max > 5.5 as strong predictors of overall survival [42].

### 3.3. Assessing Clincal Management

The impact of PET/CT in defining PC clinical management has been increasingly investigated so far. A large-scale prospective study on the role of PET/CT in PC management enrolled 550 patients; 261 of them (47%) received a diagnosis of PC after contrast-enhanced CT and subsequent PET/CT [33]. Among PC patients, PET/CT was perceived to have changed the planned management in 43% of the cases: in 11% of patients, resection was withdrawn after PET due to restaging of the disease, while in 13% of the cases surgery was planned after an initial non-surgical indication. Interestingly, when CT and PET/CT were discordant, the management suggested by PET/CT (11% vs. 4%, *p* = 0.002) was more frequently followed. Overall, the ability of PET/CT to modify clinical management varies between 30% and 44% with an estimated cost saving of $1066 per patient with an incremental PET/CT cost of $519 [36]. In a recent meta-analysis on the impact of PET/CT on patient’s management, 650 patients were included and the pooled percentage of patients who underwent management changes was 19% [36]. Interestingly, clinicians were 12 times more likely to achieve a proper diagnosis after PET/CT execution 16. While PET/CT seems to positively impact patient’s clinical management and some authors suggest its routine use in patients scheduled for surgery [36], no definitive conclusions could be drawn. Indeed, the heterogeneity of the studies and the different analyses methods (qualitative vs. quantitative) deeply impact the reported results. Due to the above-mentioned limitations, Nguyen and colleagues [34] recently hypothesized that PET/CT could be used as initial diagnostic tool to avoid futile examinations in patients with distant metastasis. Up to 20% of early-stage PC (resectable or borderline) may be down-staged preoperatively. In these cases, PET/CT fusion scans may detect occult metastases and prevent unnecessary laparotomic surgeries [43].

### 3.4. Assessing Resectability after Neoadjuvant Treatment

Patients with locally advanced or borderline resectable PC are likely to undergo NAT. In addition, selected cases of patients affected by solitary liver metastases could be included in NAT protocols to achieve resectability of the primary lesion and metastatic nodule. In this context, PET/CT could be useful to re-evaluate clinical management after NAT [32]. First, as a preliminary observation, PET/CT confirmed its ability in detecting occult metastases at CT during and after NAT. Indeed, in a retrospective study (*n* = 388), PET/CT performed during NAT localized distant metastatic lesions in 33% of patients, thus leading to the withdrawal of NAT regimen (an additional 13% of patients developed a metastatic localization soon after NAT) [16].

Another interesting perspective offered by nuclear medicine is the improvement in the evaluation of PC response to NAT, where conventional radiology shows low diagnostic potential. Indeed, in a group of borderline resectable PC patients, a low rate of response to NAT was observed using CT (1%), while an R0 resection was obtained in 95% of the cases [22]. The low ability of CT to discriminate between tumor and fibrosis could partially explain this evidence [44,45]. The ability of PET to evaluate cellular metabolism could overcome this limitation: for this reason, different studies addressed this topic and showed a correlation between SUV reduction, tumors response to NAT and post-surgical survival [16,46,47]. Yokose and colleagues [48] therefore suggested to move from RECIST (Response Evaluation Criteria in Solid tumors) criteria for CT to the adoption of PERCIST (Positron Emission Tomography Response Criteria in Solid tumors) criteria to assess preoperative response of PC to NAT using PET/CT. In their paper, the authors analyzed 22 patients with both CT and PET/CT before and after NAT. Following RECIST evaluation, partial response was defined in 9.1% of the cases while stable disease in 90.9%. Using PERCIST criteria, a higher concordance with pathological analyses was observed: 22.7% of patients had complete metabolic response, 40.9% partial metabolic response and 36.4% stable metabolic disease. In addition, in the same study, the metabolic tumor value (MTV) was associated to prognosis. Indeed, patients with > 50% reduction in MTV showed improved 1- and 3-year overall survival compared to MTV < 50% reduction (100.0% and 87.5% vs. 90% and 45%, respectively).

**Figure 1:**
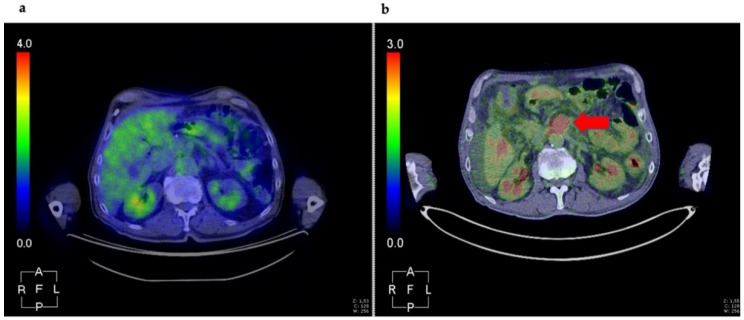
Patient diagnosed with PC (head), received six cycles of NAT (May–October 2019) and subsequent resection (December 2019). First PET/CT evaluation was performed in July 2020 for suspicious local relapse. The result was negative (**a**). During follow-up, in June 2021, a second scan revealed a preaortic mass (red arrow) compatible with disease relapse (**b**). Figure is property of the authors.

Moreover, it was suggested that metabolic activity at PET/CT could be used as a functional biomarker to guide NAT instead of CA 19.9. It is a well-known fact that CA 19.9 has a limited utility due to different confounding factors (e.g., jaundice). Conversely, ^18^F-FDG PET/CT uptake was more reliable in depicting tumor biology and it directly affects prognosis [49]. Indeed, as also demonstrated in a recent retrospective study, a decrease in metabolic parameters after NAT (*n* = 44 patients) was correlated with major pathological response and better overall survival [24]. Choi and colleagues consistently demonstrated the ability of PET/CT to discriminate NAT response and its association with improved post-surgical survival (1-year survival was 87% in responders and 28% in non-responders) [46]. Consistently with the first staging, SUV max at post-NAT PET was used to assess the risk of post-surgical recurrence: SUV max < 5 predicted an improved overall survival (SUV max < 5:42.95 months vs. SUV max > 5:26.05 months with *p* = 0.02) [50]. Worthy of note is that patients with limited response to NAT showed comparable outcome after surgery with patients who did not undergo surgery; these data point out the limited benefits of surgery in the absence of major pathological response [42]. Considering all this evidence—even though no conclusive data concerning the use of PET after NAT were published—we can hypothesize the introduction of PET/CT within restaging protocols after NAT. Figure 1 shows the clinical case of a patient resected after NAT. First PET/CT examination performed seven months after surgery for suspicious relapse described with CT scan was negative (Figure 1a), while a second examination one year later documented a preaortic mass (Figure 1b) compatible with disease relapse.

## 4. ^18^F-FDG PET Radiomics Analysis in PC

Imaging usually provides an assessment based on generic qualitative features describing the pancreatic disease. However, images contain an innumerable amount of objective data, peculiar for each patient and they might become a cornerstone of personalized medicine in the next future [51]. The scenario of quantitative image analysis has greatly improved in the last decade, providing the possibility to automatically extract and analyze several features. The term “radiomics” refers to a quantitative imaging approach, aiming to enhance the existing data available with the use of advanced mathematical analysis [52].

Radiomics analysis of morphological images has been applied to several types of cancer, whereas few studies have explored the role of radiomics applied to metabolic images obtained with PET/CT [53,54,55,56]. Research investigating the use of radiomics analysis applied to PET/CT imaging in the clinical setting of PC is mainly focused on its ability to define the predictive and/or prognostic role of this technique or to differentiate PC from other benign or precancerous conditions. In the present paragraph, the results of six studies focused on this topic are presented and discussed.

Yue et al. used radiomics evaluation of pre- and post-radiotherapy ^18^F-FDG PET/CT images to define the survival risk of patients affected by PC and the prognostic value of texture variations in predicting response to treatment. At multivariate analysis, five significant variables were identified: age, node stage, variations of homogeneity, variance and cluster tendency (*p* = 0.020, 0.040, 0.065, 0.078 and 0.081, respectively). Patients were stratified into two groups using risk score of multivariate analysis: (1) low-risk group with higher mean overall survival (29.3 months) and higher texture variation (>30%), and (2) high-risk group with reduced mean overall survival (17.7 months) and lower texture variation (<15%). Radiomics analysis of PET images seems to enhance appropriate treatment strategies for individual patients, by improving adaptive radiotherapy [57].

Similarly, Toyama and colleagues explored the prognostic value of pre-treatment ^18^F-FDG PET/CT radiomics analysis in 161 patients. Results from this study reported 10 features presenting statistical significance for overall survival prediction; however, multivariate analysis showed that grey-level zone length matrix (GLZLM) and grey-level non-uniformity (GLNU) were the only PET/CT parameter showing statistical significance. These parameters provide a representation of the heterogeneity among the pixels within a specific image, thus reflecting tumor heterogeneity. Interestingly, the combination of GLZLM, GLNU and total lesion glycolysis (TLG) was able to differentiate patients into 3 groups according to prognostic risk score [58]. Mori et al. evaluated the role of ^18^F-FDG PET radiomics in predicting distant relapse free survival (DRFS) in patients with locally advanced PC receiving radio-chemotherapy treatment. Centre of Mass Shift (COMshift) and 10th Intensity percentile (P10) were two features associated with worse prognosis, with patients presenting lower COMshift, higher P10 and having the worst outcomes in terms of DRFS. Again, radiomics analysis resulted to be a support tool in treatment personalization [59].

Lim et al. analyzed data from 48 patients with PC undergoing pre-treatment ^18^F-FDG PET to determine if gene mutations in *KRAS*, *SMAD4*, *TP53* and *CDKN2* genes were related to imaging phenotype. Alterations of *KRAS* and *SMAD4* were significantly associated with ^18^F-FDG PET/CT radiomics features. *KRAS* mutations were associated with textural features, such as long-run emphasis, zone emphasis and large-zone emphasis. These parameters belong to the Grey-level run-length Matrix, with a gray level run described as a line of pixels in a certain direction with the same intensity value. Additionally, *SMAD4* mutations showed significant correlation with SUV skewness, long-run emphasis and high-intensity textural features [60].

Zhang and colleagues carried out a radiomics analysis on ^18^F-FDG PET/CT images with the aim of assessing their ability in non-invasively differentiating autoimmune pancreatitis from PC in 111 patients. The researchers demonstrated that quantified radiomics analysis of ^18^F-FDG PET/CT images can improve the non-invasive differentiation between PC and autoimmune pancreatitis [61].

Initial explorative results from Gao et al. in 17 patients undergoing pre-treatment ^18^F-FDG PET/MRI for PC suggested that several parameters and texture features of primary PC obtained from ^18^F-FDG PET/MRI images might be useful biomarkers for predicting synchronous metastatic disease [62].

A recent metanalysis revised available studies on radiomics of the pancreas. This study concluded that radiomics is a promising quantitative imaging biomarker both of focal pancreatic lesions and diffuse changes, and that the usefulness of radiomics may vary depending on the purpose of their application. Due to the recent implementation of this process, standardization of images and image pre-processing is necessary before considering the use of radiomics of in routine clinical practice [63].

## 5. Future Directions in Preclinical PET Imaging for PC

^18^F-FDG is the commonly used tracer in clinical practice for PC. Beyond ^18^F-FDG, many other molecular targets, suitable for tracer development, have been investigated in preclinical models of PC [64], primarily focusing on the targets acting in the process that involves the progression from pancreatic intraepithelial neoplasia (PanIN) into PC [65]. Herein, we will offer an overview of the most recent PET/CT tracers (and targets) that have been investigated in animal models of PC (Table 2) [64,66].

Integrins are a large family of heterodimeric transmembrane proteins mediating cell interactions. Integrin tracer technology uses peptides containing sequences that preferentially bind to integrin αvβ3, αvβ5 and αvβ6, the latter being mostly over-expressed in PC [67]. Ui et al. [68] developed [^68^Ga]Ac-CG6, an imaging probe for PET/CT targeting αvβ6 integrin-positive PC. [^68^Ga]Ac-CG6 showed an intratumoral distribution reflecting the αvβ6 integrin-positive regions detected by immunohistochemistry and resulted in an increase in tumor uptake with parallel decrease in non-specific accumulation in the liver, spleen and kidneys. Another αvβ6 integrin tracer, [^68^Ga]-DOTA-SFLAP3, was developed by Müller et al. [72] and tested preclinically and in one patient. In the animal model, the tracer showed considerable accumulation in the tumor with a relatively whole-body fast clearance; PET/CT imaging in the patient allowed detection of pancreatic metastases with favorable dosimetric values and high tumor-to-background contrast, warranting further investigation as a potential diagnostic tracer. Another integrin, α3β1, was also recently investigated as a target by Li et al. [69]; [^68^Ga]NOTA-CK11 showed high tumor uptake with a good tumor-to-blood ratio and tumor-to-muscle ratio. In addition, tumor accumulation was significantly higher when compared to the unlabeled CK11 injection group. The clinical relevance of targeting integrins is that it is undetectable in healthy adult epithelium, but significantly up-regulated in a wide range of epithelial derived cancers.

**Figure 2:**
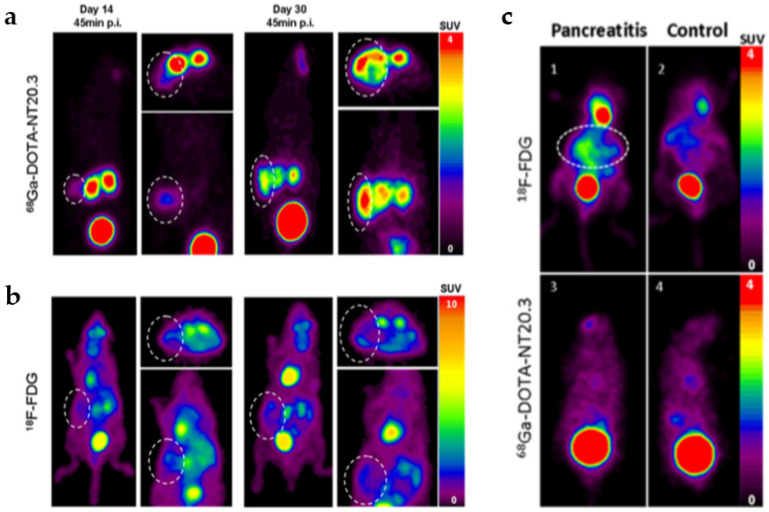
PET imaging 45 min post injection of [^68^Ga]DOTA-NT-20.3 (**a**) and ^18^F-FDG (**b**) in an orthotopic PC model. Circles delineate the tumors. Uptake by normal tissues was observed with ^18^F-FDG (**b**) compared to the low uptake obtained with ^68^[Ga]DOTA-NT-20.3 (**a**). In vivo PET images of mice with pancreatitis and control mice with ^18^F-FDG and [^68^Ga]DOTA-NT-20.3 (**c**) In mice with acute pancreatitis, ^18^F-FDG uptake was elevated, in contrast with low uptake in control mice (**c**, **upper panel**). [^68^Ga]DOTA-NT-20.3 PET imaging did not show radiotracer uptake in any case (**c**, **bottom panel**), reprinted with permission from Ref [74]. Copyright 2019 Copyright American Chemical Society (ACS).

Another investigated target is Neurotensin, a peptide normally present in the gastrointestinal tract and the brain. It triggers a wide variety of functions through interaction with, among others, the neurotensins receptor 1 (NTSR1), which is known to be over-expressed in PC tumor, as well as in high-grade PanINs [73]. Research reported the use of the ^68^Ga-labeled NTS analogue DOTA-NT-20.3 in human cancer animal models and to discriminate PC from pancreatitis [74]. Dynamic PET/CT imaging with [^68^Ga]DOTA-NT-20.3 showed focal uptake in subcutaneous and orthotopic tumors and high-quality images in other organs with high specificity of the tracer. Moreover, authors compared [^68^Ga]DOTA-NT-20.3 and ^18^F-FDG PET/CT imaging in the orthotopic xenograft to an experimental acute pancreatitis. They found low uptake of ^18^F-FDG in orthotopic tumors. In contrast, tumor on PET images with [^68^Ga]DOTA-NT20.3 was well detected (Figure 2a,b). Furthermore, high tumor selectivity and low uptake in pancreatic inflammation support the potential clinical use of [^68^Ga]DOTA-NT20.3 to discriminate PC from pancreatitis (Figure 2c). Other diagnostic applications include the possibility of using this radiotracer as a theragnostic agent aimed at selecting patients with a non-resectable PC and using a [^177^Lu]NTSR1 targeting ligand as regards radiotherapy. Another tracer targeting NTS1 developed by Wang et al. [75] demonstrated a higher tumor uptake in the animal model expressing lower NTR1 level compared to a different PC model where NTR1 expression was higher. The discrepancy might be caused by different levels of microvascular density, and therefore PET/CT scans may not reflect the expression since blood supply would affect to the tumor uptake. Not only NTR-targeted PET/CT may be used to diagnose NTR1 positive PC but also it may be fully integrated to NTR target therapy.

**Figure 3:**
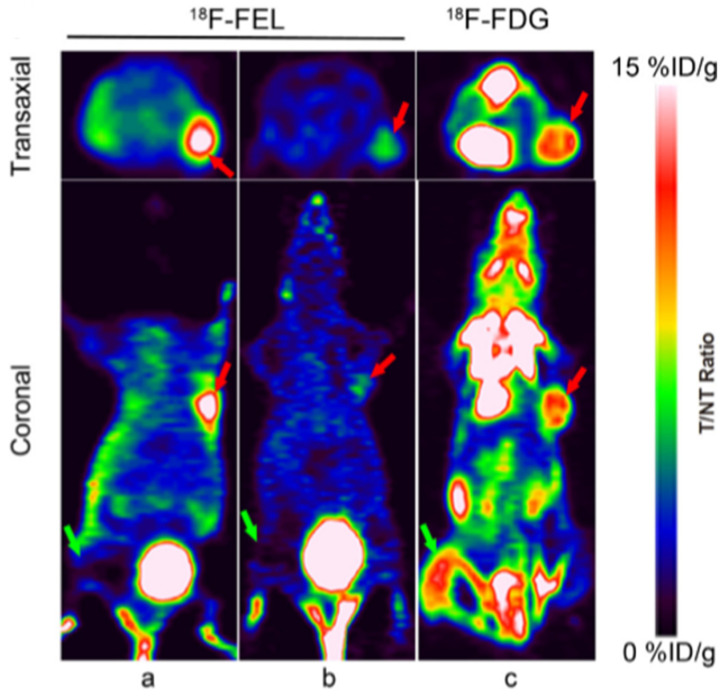
PET imaging of T3M4 tumors-bearing and inflammation nude mice 60 min after injection of ^18^F-FEL (**a**) and ^18^F-FDG (**c**). PET images 60 min after co-injection of β-D-lactose as competitor (**b**). Tumors are marked by red arrows and inflammatory lesions are marked by green arrows. Tumors were delineated 60 min after injection of ^18^F-FEL, while low background and absence of accumulation was present in inflammatory tissue. Reprinted with permission from Ref [78]. Copyright 2017 Copyright Shaobo Yao, et al.

HIP/PAP (Hepatocarcinomaintestine-pancreas and pancreatitis-associated protein) is a secreted plasma protein, also known as “lactose-binding protein” [76]. The over-expression of HIP/PAP is linked to many different diseases including PC [70,77]. Yao et al. [78] synthesized 1′-^18^F-fluoroethyl-β-D-lactose ([^18^F]FEL) which exhibited high/noteworthy HIP/PAP-binding affinity, stability, and specific tumor accumulation in pancreatic tumors in comparison with ^18^F-FDG. More interestingly, the same tumor-bearing mice were also intramuscular injected with turpentine as models of aseptic inflammation. Results from the study showed that the tumor-to-muscle, tumor-to-blood and tumor-to-inflammation ratio for ^18^F-FDG were reduced with respect to those found for [^18^F]FEL (Figure 3a,c). However, HIP/PAP is also overexpressed in pancreatic cells compared to normal pancreas in condition of pancreatitis [79,80]. Therefore, the ability of [^18^F]FEL to differentiate neoplastic vs. inflammatory pancreatic diseases should be further determined using genetically engineered mouse models, indeed the tracer has this clinical potential and advantage over ^18^F-FDG.

Finally, Tissue factor (TF), over-expressed in PC, serves as the primary initiator of the extrinsic pathway of blood coagulation [79]. There is a strong correlation between the aberrant expression of TF, staging and overall survival in PC [71,81]. Similarly, CD105 is a proliferation-associated cell-surface protein highly expressed on activated endothelial cells, and its over-expression is associated with decreased patient survival for most cancers, and in particular for PC [82]. Luo et al. [83] developed a novel bispecific heterodimer, targeting TF and CD105 to recognize early disease features and monitor therapeutic response. They observed a significantly enhanced tumor uptake when using the 64Cu-labeled heterodimer in orthotopic tumor-bearing mice compared to each of their monospecific Fab tracers. Even if the heterodimer was shown to be a more effective PET imaging agent in comparison to single-targeted antibody tracers, the clinical benefits of heterodimeric imaging agents in patients have been unexplored and need further evaluation.

## 6. Conclusions

PET may play a role in diagnosis and treatment of resectable PC and its use should be considered on a case-by-case basis. Moreover, its use is likely to be cost-effective in the clinical management of PC, preventing unnecessary multiple examinations. Despite this, PET is not recommended by international guidelines in the first instance and its role in the management of PC is still a matter of debate.

## Figures and Tables

**Table 1:** NCCN, ESMO and AIOM guidelines for the use of CT, MRI and PET in PC.

Guidelines	Version	CT	MRI	^18^F-FDG PET/TC
NCCN [17]	1.2021	Recommended for Initial Diagnosis	Recommended for Problem Solving	Not Univocally Defined
		CT of abdomen preferred. MDCT angiography with sub-millimeter, axial sections (pancreatic and portal venous phases) is the preferred imaging tool	RMN preferred as a problem-solving tool, particularly for characterization of CT-indeterminate liver lesion and when suspected PC is not visible on CT.	Role of PET/CT remains unclear. PET scan may be considered after formal pancreatic CT protocol in high-risk patients (borderline resectable disease, markedly elevated CA 19.9, large primary tumor, large regional nodes) to detect extra-pancreatic metastases. Not substitute for high-quality CT.
ESMO [18]	2015	Recommended for Initial Diagnosis	Recommended for Problem Solving	Not Univocally Defined
		Radiological studies should include CT angiography at the pancreatic arterial (40–50 s) and portal venous (65–70 s) phases	When assessing vessel involvement, use of MRI left to expert discretion. It shows equal benefit to CT with no superiority. MRI is useful for detection of hepatic lesions that cannot be characterized by CT	PET/CT does not add staging information in resectable disease and cannot be recommended.
AIOM [19]	2020	Recommended for Initial Diagnosis	Recommended for Problem Solving (Liver)	Not Reported
		In patients with suspected PC, multislice CT scan of the chest and abdomen is the first-choice	MRI improves liver staging in patients with potentially resectable PC	

Legend: CA 19.9: carbohydrate antigen 19.9; CT: computed tomography; MDCT: multidetector computed tomography; MRI: magnetic resonance imaging; PC: pancreatic cancer; PET/CT: positron emission tomography/computed tomography.

**Table 2:** List of PET tracers (and targets) investigated in animal models of PC.

Molecular Target	Tracer	Animal Model	Clinical Relevance	Reference
Integrin α_v_β_6_	[^68^Ga]Ac-CG6	AsPC-1 MIA PaCa-2	Specific affinity to α_v_β_6_	Ui T, Bioorganic and Medicinal Chemistry, 2020 [66]
Integrin α_v_β_6_	[^68^Ga]DOTA-SFLAP3	Capan-2	Specific affinity to α_v_β_6_	Muller M, Nuklearmedizin 2019 [67]
Integrin α_3_β_1_	[^68^Ga]NOTA-CK11	SW1990	Specific affinity to α_3_β_1_	Li H, Mol Pharm 2020 [68]
Neurotensin receptor 1 (NTS1)	[^68^Ga]DOTA-NT20.3	AsPC-1	Differential diagnosis for tumour and not for inflammatory lesion	Prignon A, Mol Pharm 2019 [69]
Hepatocarcinomaintestine-pancreas and pancreatitis-associated protein (HIP/PAP)	[^18^F]FEL	T3M4	Differentiating tumors from aseptic inflammation	Yao S, Oncotarget 2017 [70]
Tissue factor (TF) CD105	[^64^Cu]-NOTA-heterodimer	BxPC-3 (TF/CD105^+/+^)	Specific affinity to TF and CD105	Luo H, Clin Cancer Res. 2016 [71]

## Data Availability

No new data were created or analyzed in this study. Data sharing is not applicable to this article.
